# Travel-Related Diagnoses Among U.S. Nonmigrant Travelers or Migrants Presenting to U.S. GeoSentinel Sites — GeoSentinel Network, 2012–2021

**DOI:** 10.15585/mmwr.ss7207a1

**Published:** 2023-06-30

**Authors:** Ashley B. Brown, Charles Miller, Davidson H. Hamer, Phyllis Kozarsky, Michael Libman, Ralph Huits, Aisha Rizwan, Hannah Emetulu, Jesse Waggoner, Lin H. Chen, Daniel T. Leung, Daniel Bourque, Bradley A. Connor, Carmelo Licitra, Kristina M. Angelo

**Affiliations:** ^1^Division of Global Migration and Quarantine, National Center for Emerging and Zoonotic Infectious Disease, CDC; ^2^Department of Global Health, Boston University School of Public Health, Boston, Massachusetts; ^3^Section of Infectious Disease, Department of Medicine, Boston University Chobanian & Avedisian School of Medicine, Boston, Massachusetts; ^4^Division of Infectious Diseases (Emerita), Department of Medicine, Emory University, Atlanta, Georgia; ^5^J.D. MacLean Centre for Tropical Diseases, McGill University, Montreal, Canada; ^6^Department of Infectious Tropical Diseases and Microbiology, IRCCS Sacro Cuore Don Calabria Hospital, Negrar, Verona, Italy; ^7^GeoSentinel, International Society of Travel Medicine, Alpharetta, Georgia; ^8^Department of Medicine, Emory University School of Medicine, Atlanta, Georgia; ^9^Department of Medicine, Mount Auburn Hospital, Cambridge, Massachusetts; ^10^Harvard Medical School, Boston, Massachusetts; ^11^Division of Infectious Diseases, University of Utah School of Medicine, Salt Lake City, Utah; ^12^Department of Psychology, Colorado State University, Fort Collins, Colorado; ^13^Infectious Diseases, Orlando Health Medical Group, Orlando, Florida

## Abstract

**Problem/Condition:**

During 2012–2021, the volume of international travel reached record highs and lows. This period also was marked by the emergence or large outbreaks of multiple infectious diseases (e.g., Zika virus, yellow fever, and COVID-19). Over time, the growing ease and increased frequency of travel has resulted in the unprecedented global spread of infectious diseases. Detecting infectious diseases and other diagnoses among travelers can serve as sentinel surveillance for new or emerging pathogens and provide information to improve case identification, clinical management, and public health prevention and response.

**Reporting Period:**

2012–2021.

**Description of System:**

Established in 1995, the GeoSentinel Network (GeoSentinel), a collaboration between CDC and the International Society of Travel Medicine, is a global, clinical-care–based surveillance and research network of travel and tropical medicine sites that monitors infectious diseases and other adverse health events that affect international travelers. GeoSentinel comprises 71 sites in 29 countries where clinicians diagnose illnesses and collect demographic, clinical, and travel-related information about diseases and illnesses acquired during travel using a standardized report form. Data are collected electronically via a secure CDC database, and daily reports are generated for assistance in detecting sentinel events (i.e., unusual patterns or clusters of disease). GeoSentinel sites collaborate to report disease or population-specific findings through retrospective database analyses and the collection of supplemental data to fill specific knowledge gaps. GeoSentinel also serves as a communications network by using internal notifications, ProMed alerts, and peer-reviewed publications to alert clinicians and public health professionals about global outbreaks and events that might affect travelers. This report summarizes data from 20 U.S. GeoSentinel sites and reports on the detection of three worldwide events that demonstrate GeoSentinel’s notification capability.

**Results:**

During 2012–2021, data were collected by all GeoSentinel sites on approximately 200,000 patients who had approximately 244,000 confirmed or probable travel-related diagnoses. Twenty GeoSentinel sites from the United States contributed records during the 10-year surveillance period, submitting data on 18,336 patients, of which 17,389 lived in the United States and were evaluated by a clinician at a U.S. site after travel. Of those patients, 7,530 (43.3%) were recent migrants to the United States, and 9,859 (56.7%) were returning nonmigrant travelers.

Among the recent migrants to the United States, the median age was 28.5 years (range = <19 years to 93 years); 47.3% were female, and 6.0% were U.S. citizens. A majority (89.8%) were seen as outpatients, and among 4,672 migrants with information available, 4,148 (88.8%) did not receive pretravel health information. Of 13,986 diagnoses among migrants, the most frequent were vitamin D deficiency (20.2%), *Blastocystis* (10.9%), and latent tuberculosis (10.3%). Malaria was diagnosed in 54 (<1%) migrants. Of the 26 migrants diagnosed with malaria for whom pretravel information was known, 88.5% did not receive pretravel health information. Before November 16, 2018, patients’ reasons for travel, exposure country, and exposure region were not linked to an individual diagnosis. Thus, results of these data from January 1, 2012, to November 15, 2018 (early period), and from November 16, 2018, to December 31, 2021 (later period), are reported separately. During the early and later periods, the most frequent regions of exposure were Sub-Saharan Africa (22.7% and 26.2%, respectively), the Caribbean (21.3% and 8.4%, respectively), Central America (13.4% and 27.6%, respectively), and South East Asia (13.1% and 16.9%, respectively). Migrants with diagnosed malaria were most frequently exposed in Sub-Saharan Africa (89.3% and 100%, respectively).

Among nonmigrant travelers returning to the United States, the median age was 37 years (range = <19 years to 96 years); 55.7% were female, 75.3% were born in the United States, and 89.4% were U.S. citizens. A majority (90.6%) were seen as outpatients, and of 8,967 nonmigrant travelers with available information, 5,878 (65.6%) did not receive pretravel health information. Of 11,987 diagnoses, the most frequent were related to the gastrointestinal system (5,173; 43.2%). The most frequent diagnoses among nonmigrant travelers were acute diarrhea (16.9%), viral syndrome (4.9%), and irritable bowel syndrome (4.1%).

Malaria was diagnosed in 421 (3.5%) nonmigrant travelers. During the early (January 1, 2012, to November 15, 2018) and later (November 16, 2018, to December 31, 2021) periods, the most frequent reasons for travel among nonmigrant travelers were tourism (44.8% and 53.6%, respectively), travelers visiting friends and relatives (VFRs) (22.0% and 21.4%, respectively), business (13.4% and 12.3%, respectively), and missionary or humanitarian aid (13.1% and 6.2%, respectively). The most frequent regions of exposure for any diagnosis among nonmigrant travelers during the early and later period were Central America (19.2% and 17.3%, respectively), Sub-Saharan Africa (17.7% and 25.5%, respectively), the Caribbean (13.0% and 10.9%, respectively), and South East Asia (10.4% and 11.2%, respectively).

Nonmigrant travelers who had malaria diagnosed were most frequently exposed in Sub-Saharan Africa (88.6% and 95.9% during the early and later period, respectively) and VFRs (70.3% and 57.9%, respectively). Among VFRs with malaria, a majority did not receive pretravel health information (70.2% and 83.3%, respectively) or take malaria chemoprophylaxis (88.3% and 100%, respectively).

**Interpretation:**

Among ill U.S. travelers evaluated at U.S. GeoSentinel sites after travel, the majority were nonmigrant travelers who most frequently received a gastrointestinal disease diagnosis, implying that persons from the United States traveling internationally might be exposed to contaminated food and water. Migrants most frequently received diagnoses of conditions such as vitamin D deficiency and latent tuberculosis, which might result from adverse circumstances before and during migration (e.g., malnutrition and food insecurity, limited access to adequate sanitation and hygiene, and crowded housing,). Malaria was diagnosed in both migrants and nonmigrant travelers, and only a limited number reported taking malaria chemoprophylaxis, which might be attributed to both barriers to acquiring pretravel health care (especially for VFRs) and lack of prevention practices (e.g., insect repellant use) during travel. The number of ill travelers evaluated by U.S. GeoSentinel sites after travel decreased in 2020 and 2021 compared with previous years because of the COVID-19 pandemic and associated travel restrictions. GeoSentinel detected limited cases of COVID-19 and did not detect any sentinel cases early in the pandemic because of the lack of global diagnostic testing capacity.

**Public Health Action:**

The findings in this report describe the scope of health-related conditions that migrants and returning nonmigrant travelers to the United States acquired, illustrating risk for acquiring illnesses during travel. In addition, certain travelers do not seek pretravel health care, even when traveling to areas in which high-risk, preventable diseases are endemic. Health care professionals can aid international travelers by providing evaluations and destination-specific advice.

Health care professionals should both foster trust and enhance pretravel prevention messaging for VFRs, a group known to have a higher incidence of serious diseases after travel (e.g., malaria and enteric fever). Health care professionals should continue to advocate for medical care in underserved populations (e.g., VFRs and migrants) to prevent disease progression, reactivation, and potential spread to and within vulnerable populations. Because both travel and infectious diseases evolve, public health professionals should explore ways to enhance the detection of emerging diseases that might not be captured by current surveillance systems that are not site based.

## Introduction

Modern modes of transportation and growing economies have made traveling more efficient and accessible. This progress has resulted in a surge of international travel, including travel to remote destinations and lower-income countries (*1*). In 2019, a record 2.4 billion international tourist arrivals globally (*2*) were observed by the World Tourism Organization.

Four studies estimated that 43%–79% of travelers to low- and middle-income countries became ill with a travel-related health problem, some of whom needed medical care during or after travel (*3*). Certain groups (e.g., travelers visiting friends and relatives [VFRs] and migrants) are particularly at risk for acquiring travel-related diseases because of a lack of risk awareness, access to specialized health care and pretravel consultation, and trust in the health care system (*4*,*5*). In addition, travelers might introduce pathogens into new environments and populations, leading to the spread of novel and emerging infectious diseases (*6*). The 2019 measles outbreaks across Europe illustrated how travel and poor vaccination coverage among local populations can fuel an epidemic (*7*). These outbreaks resulted in the importation of measles to communities with low vaccination coverage in the United States, a country that had eliminated measles in 2000. The rapid spread of disease across international borders also was observed during the Ebola virus disease epidemic in West Africa during 2014–2016 (*8*) as well as during the COVID-19 pandemic (*9*). These events illustrate the dangers of introducing pathogens into geographic clusters of susceptible populations as well as the importance of vaccination and other preventative strategies to reduce the risk for importation and spread.

Studying illness among travelers improves case identification, clinical management, and public health prevention strategies and also helps to characterize the epidemiology of diseases and control their spread (*10*). Because international travel continues to increase, conducting surveillance and research regarding travel-related diseases will be instrumental in reducing global transmission. To identify travel-related diseases and facilitate rapid communication between clinicians and public health professionals globally, a surveillance system (e.g., GeoSentinel) is needed. Such connectivity can reduce the size of outbreaks while promoting the timely sharing of clinical insight regarding the diagnosis and treatment of patients.

The GeoSentinel Network (GeoSentinel) is a global, clinical-care–based surveillance and research network of travel and tropical medicine sites that monitors infectious diseases and other adverse health events that affect international travelers (https://geosentinel.org/). Since its inception in 1995, GeoSentinel has remained at the forefront of travel-related sentinel surveillance and continues to refine its collection of epidemiologic data from ill travelers during and after travel.

This report describes GeoSentinel, key changes in its data collection, its successful detection of sentinel events, and future directions. This report also summarizes the data collected from migrants and returning U.S. nonmigrant travelers presenting for evaluation at a U.S. GeoSentinel site during 2012–2021. The findings in this report underscore the importance of global travel-related disease surveillance so that clinicians and public health professionals are aware of the most common travel-related illnesses and can develop improved treatment and prevention strategies.

## Methods

### The GeoSentinel Network

#### Overview

GeoSentinel is a collaboration between CDC and the International Society of Travel Medicine (ISTM) and was established in the United States in 1995 with nine U.S. sites (*11*). During 1996–1997, the GeoSentinel network expanded globally. GeoSentinel’s primary purpose is to coordinate multiple clinical-care–based sites that operate a global, provider-based emerging infections sentinel network, conduct surveillance for travel-related infections, and communicate and help guide public health responses (*12*). Sites collaborate to report disease or population-specific findings through retrospective database analyses and the collection of supplemental data to fill specific knowledge gaps.

#### Sites and Affiliate Members

**FIGURE 1:**
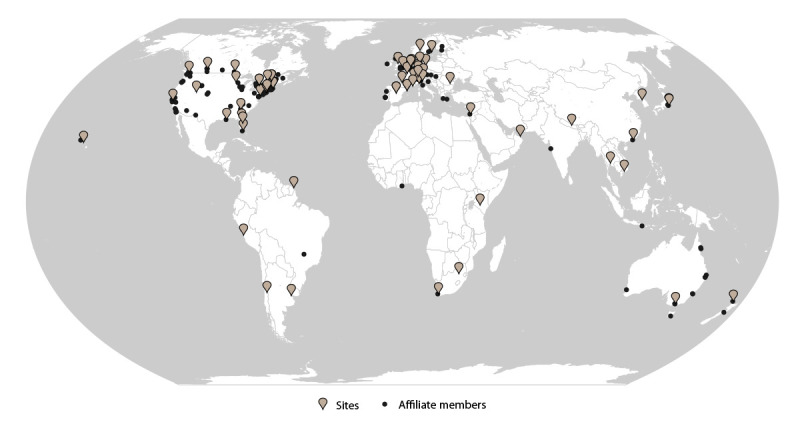
GeoSentinel sites and affiliate members — GeoSentinel Network, 2012–2021* * Sites = 71; affiliate members = 164.

As of December 2021, GeoSentinel comprised 71 sites in 29 countries located on six continents (Figure 1). GeoSentinel sites are health care facilities led by site directors and codirectors who are medical professionals with expertise in travel and tropical medicine. GeoSentinel also includes 164 affiliate members (formerly referred to as network members) who report sentinel or unusual travel medicine cases but do not enter data into the GeoSentinel database.

#### Eligible Patients

Patient data can be entered into the GeoSentinel database if the patient has crossed an international border and was seen at a GeoSentinel site with a possible travel-related illness or, in the case of certain migrants, for screening purposes upon entry into their arrival country. Data from patients who develop a complication from pretravel treatments (i.e., adverse effect from vaccinations or antimalarial medication) also might be entered, even if the patients have not yet departed on their trip.

#### Data Collection

GeoSentinel sites use a standardized data collection form (Supplementary Appendix, https://stacks.cdc.gov/view/cdc/127681) to collect demographic, clinical, and travel-related information about patients and the illnesses acquired during travel. These data are collected electronically via a secure web-based data entry application based at CDC. Daily reports are generated for assistance in detecting events (i.e., unusual patterns or clusters of disease). The system emails these reports to both CDC and ISTM partners for review. If an unusual disease pattern or cluster of disease is detected, the GeoSentinel program manager sends an email to the site requesting additional information. Electronic validation is integrated into the database to reduce data entry errors and maintain data integrity. Whereas certain sites enter all travel-related cases into the GeoSentinel database, other sites only enter a convenience sample. Entry of cases into the GeoSentinel database and determination of travel association are at the discretion of the treating clinician. This activity was reviewed by CDC and was conducted consistent with applicable federal law and CDC policy.* Ethics clearance has been obtained by sites as required by their respective institutions.

### Selected Variables and Definitions

The GeoSentinel database contains information obtained from patients evaluated at GeoSentinel sites during and after international travel. The following definitions were used during the study period.

**Citizen.** A person who is a legally recognized national of a country.

**Clinical setting.** The timing of the visit related to travel.

- During travel. The trip related to the current illness is in progress. This category includes expatriates seen in their country of residence for illnesses likely acquired in that country or where the country of exposure cannot be ascertained.
- After travel. The trip related to the current illness has been completed. This category also includes expatriates who acquire an illness during travel outside their current country of residence and where the relevant exposure is related to travel.

**Diagnosis and diagnosis type.** Site directors choose from approximately 475 diagnoses classified as either etiologic or syndromic. A write-in option is available on the data collection form if the diagnosis is not on the list.

- Etiologic. This diagnosis type reflects a specific disease. The “diagnosis status” of etiologic diagnoses might be “confirmed” or “probable” (see Diagnosis status).
- Syndromic. This diagnosis type reflects symptom- or syndrome-based etiologies when a more specific etiology is not known or could not be determined as a result of use of empiric therapy, self-limited disease, or inability to justify additional diagnostic tests beyond standard clinical practice. The “diagnosis status” of all syndromic diagnoses is “confirmed” (see Diagnosis status).

**Diagnosis status**. The diagnosis is categorized in one of two ways on the basis of available diagnostic methods:

- Confirmed. The diagnosis has been made by an indisputable clinical finding (e.g., removal of larvae of tungiasis) or diagnostic test.
- Probable. The diagnosis is supported by evidence (including diagnostic testing) strong enough to establish presumption but not proof.

**Expatriate.** A person living in a destination with an independent residence and address and using the same infrastructure as local residents of the same economic class. Expatriates intend to remain in-country for ≥6 months and have no intention to legally change their citizenship or permanent residency status.

**Main symptoms.** The symptoms associated with the illness that was the reason for the clinic visit.

**Migrant.** A person who, at some time in their life, has emigrated from their country of birth and has previously or intends to legally change their citizenship or permanent residency status. The resident country is entered on the data collection form as the new home country.

**Nonmigrant traveler.** A person who is traveling for a purpose unrelated to migration.

**Pretravel encounter.** Any pretravel health visit or the receipt of travel-related health information.

**Resident.** A person who has their primary residence in a particular country.

**Severity.** The highest level of clinical care received for the travel-related diagnosis, including outpatient, inpatient ward, and inpatient intensive care unit (ICU) care.

**Syndrome or system groupings of diagnoses.** All GeoSentinel diagnoses are categorized into groups according to the type of syndrome or system affected (Box 1).

BOX 1 **Syndrome and system groupings of diagnoses for surveillance — GeoSentinel Network, 2012–2021**

- Adverse events to medication or vaccine
- Animal bites or scratches
- Death
- Dermatological: infectious or potentially travel related
- Dermatological: preexisting or chronic disease or comorbidity
- Febrile or systemic syndrome
- Gastrointestinal: infectious or potentially travel related
- Gastrointestinal: preexisting or chronic disease or comorbidity
- Genitourinary and STDs: infectious or potentially travel related
- Genitourinary and STDs: pre-existing or chronic disease or comorbidity
- Musculoskeletal: infectious or potentially travel related
- Musculoskeletal: pre-existing or chronic disease or comorbidity
- Neurological: infectious or potentially travel related
- Neurological: preexisting or chronic disease or comorbidity
- Other:* infectious or potentially travel related
- Other:* chronic disease or comorbidity
- Respiratory or ENT: infectious or potentially travel related
- Respiratory or ENT: pre-existing or chronic disease or comorbidity
- Screening

**Abbreviations:** ENT = ears, nose, and throat; STD = sexually transmitted disease.

* Diseases that do not fall into system groupings.

**Travel reason.** Primary reason for travel related to the current illness (Box 2).

BOX 2 **Reason for travel — GeoSentinel Network, 2012–2021**

- **Tourism (vacation):** Includes all travel for tourism or leisure. Also includes travel that might involve visiting friends and relatives overseas if the traveler is not a first- or second-generation immigrant returning to his or her country of origin.
- **Business or occupational**
  **° Conference:** Travel by an employed person for the purpose of attending a conference or convention
  **° Corporate or professional:** Travel by an employed person for the purpose of carrying out business, attending meetings, or other work-related events
  **° Business or occupational — research:** Travel by an employed person for the purpose of field work, laboratory work, or other type of academic research
  **° Business or occupational — other:** Travel for the purpose of business or as part of one’s occupation but where the travel does not fit in the other specific categories of research, study, conference, or seasonal migrant work
- **Seasonal or temporary work (migrant worker):** Travel for the purpose of pursuing seasonal or other nonpermanent work because of economic opportunities in countries other than the person’s country of birth or place or permanent residence. These persons usually do not have any intention or permission to stay permanently in the country or region in which they are working.
- **Student:** Travel by a student for the purpose of study abroad, attending a student conference, research, or other educational purpose
- **Migration:** Main reason for travel is intent or need to resettle outside of birth country or country of secondary migration
- **Providing medical care:** Travel for the purpose of providing medical care
- **VFR:** Person is traveling from the region in which they are currently residing (usually as a migrant, expatriate, or long-term visitor) to their region of origin (e.g., a low-income country) to visit friends and relatives. This reason for travel includes persons who are travelling with a child/grandchild (second-generation VFRs) or parent and those traveling with a spouse or partner.
- **Military:** Main purpose is deployment to the country visited or to participate in military operations
- **Missionary, humanitarian aid, volunteer, or community service:** Travel to perform humanitarian work, community service, or take part in volunteer work (includes travel prompted by participation in a religious organization). If the purpose is primarily to provide health care, then the reason for travel should instead be providing medical care.
- **Retirement:** Travel for the purpose of retiring to a new location. Certain of these persons will be expatriates or long-term visitors.
- **Planned medical care**: Main purpose of travel is to obtain medical care
- **Not ascertainable:** Reason for travel cannot be ascertained or is unknown

**Abbreviation:** VFR = visiting friends and relatives.

**Travel related.** Designates the relation of the main diagnosis to the patient’s travel.

- Travel related. Used when the illness under evaluation, initially suspected to be travel-related, was determined to have been acquired during the patient’s travel.
- Imported infection. Used for infections acquired in the patient’s country of residence if exported to another country and then evaluated at a GeoSentinel site.
- Not travel related. Used when the illness under evaluation, initially suspected to be travel related, was determined to have been acquired before departure from or after returning to the home country.
- Not ascertainable*.* Used when the illness under evaluation, initially suspected to be travel related, was equally likely to have been acquired during the patient’s travel or before departing from or after returning to the residence country.

### Changes to GeoSentinel Data Collection

During 2012–2021, multiple changes were made to the GeoSentinel data entry application (Box 3). New fields and subfields that collect detailed information on patient types, diagnoses, and trip information were added to provide a complete profile of patients and their associated illnesses. Additional fields were added for diseases of interest to provide information (e.g., vaccination status, etiology [e.g., organism genus and species], and cause of death). Case definitions were developed for each diagnosis code, and data collection fields were refined on an ongoing basis to aid clinicians in classifying patients and diagnoses.

BOX 3 **Changes to the GeoSentinel data entry application — GeoSentinel Network, 2012–2021**

**March 2013**

- Added date of illness onset
- Added preexisting conditions (e.g., HIV, cancer, or diabetes), including use of immunosuppressive drugs
- Added a requirement to mark a “primary diagnosis” if more than one diagnosis was entered
- Added new fields for diagnosis activity (active or resolved) and if diagnosed by screening

**October 2015**

- Modified function for “complete” records to include only those with infectious diagnoses or those that were travel related
- Added fields to capture the highest level of care required for the illness (severity), where the patient obtained pretravel information, and a write-in field for general comments
- Modified main presenting symptoms
- Updated reason for travel options
- Added fields for activities during travel, including
  **°** Animal exposure
  **°** Antibiotic taken during travel
  **°** Attended mass gathering
  **°** Blood or body fluid exposure
  **°** Provided medical care
  **°** Staying or eating in local homes
  **°** Unplanned medical or dental care
- Added ability to capture diagnosis method(s)
- Created supplemental data form to collect antibiotic resistance data on nine pathogens (*Campylobacter* spp., *Escherichia coli*, *Klebsiella pneumoniae*, *Salmonella* spp., *S. enterica* Typhi, *S. enterica* Paratyphi, *Shigella* spp., *Staphylococcus aureus*, and *Streptococcus pneumoniae*)
- Initiated special projects for mass gatherings and rabies postexposure prophylaxis

**October 2016**

- Modified main presenting symptoms and diagnostic methods
- Added ability to collect specimen type and organism genus and species
- Added geographic alerts for certain diseases (Barmah Forest virus, chronic Chagas disease, coccidioidomycosis, filariasis, malaria, paracoccidioidomycosis, Ross River virus, and schistosomiasis) that are reported from unexpected countries and regions
- Added required additional information for certain diseases, including vaccination status, etiology (e.g., organism genus and species), and cause of death
- Deployed enhanced surveillance migrant form to capture detailed information on migrants

**November 2017**

- Modified main presenting symptoms
- Added subcategories for VFRs, identifying the VFR as the person, child or dependent, or spouse or partner
- Added option for secondary reason for travel and country of exposure for VFRs
- Added additional questions for certain diagnoses (i.e., malaria, leishmaniasis, and Zika)
- Added QA alerts to ensure that certain diagnoses meeting the case definition using the diagnosis methods to be marked confirmed
- Began collecting data for enhanced surveillance projects for rickettsioses, planned and unplanned healthcare abroad

**August 2018**

- Updated production database from Microsoft SQL Server 2008 to Microsoft SQL Server 2016

**November 2018**

- Combined supplemental migrant data collection form with main data collection form
- Updated expatriate and long-term visitor definitions and added subcategory options
- Added reason for travel, country of exposure, and region of exposure fields to each diagnosis
- Added imported infection as a travel-related option for migrants
- Modified main presenting symptoms

**April 2019**

- Added new project for respiratory illness in older travelers

**October 2019**

- Removed variables for primary diagnosis and patient type fields (inpatient, outpatient, tele-consult inpatient, and tele-consult outpatient)
- Removed student subchoices for travel reason field
- Added field for required medical evacuation
- Updated antibiotic resistance drug options
- Modified main presenting symptoms
- Revised antibody diagnosis method to specify whether IgM or IgG

**March 2020**

- Deployed enhanced surveillance project for respiratory illness in travelers related to COVID-19

**August 2020**

- Deployed enhanced surveillance project for sentinel identification of respiratory illness in travelers related to COVID-19

**November 2021**

- Updated COVID-19 vaccination status, including boosters

**Abbreviations:** IgG = immunoglobulin G; IgM = immunoglobulin M; QA = quality assurance; VFR = visiting friends and relatives.

Internal validation is now used to ensure that data are collected uniformly and accurately among sites. The collection of diagnostic methods allows for validation of confirmed and probable cases, and quality assurance (QA) alerts prevent sites from classifying diagnoses as confirmed during data entry without required disease-specific diagnostic methods. Other QA alerts prevent the skipping of required fields as well as logical errors.

Before November 16, 2018, the variables of travel reason and exposure country (and region) were not linked to an individual diagnosis. Instances where patients had multiple unrelated diagnoses made it difficult to ascertain what information applied to which diagnosis. As a result, the data collection form and database were updated to specify travel reason and exposure country information for each individual diagnosis.

To fill knowledge gaps, enhanced surveillance projects were deployed throughout the analysis period to collect specific information about a disease or types of travelers that was not collected on the core data collection form. This included projects on antibiotic resistance for selected bacterial pathogens, rickettsioses, mass gatherings (*13*), rabies postexposure prophylaxis (*14*), planned and unplanned health care abroad (*15*), migrants (*16*), and respiratory illnesses related to COVID-19.

## Analysis

**FIGURE 2:**
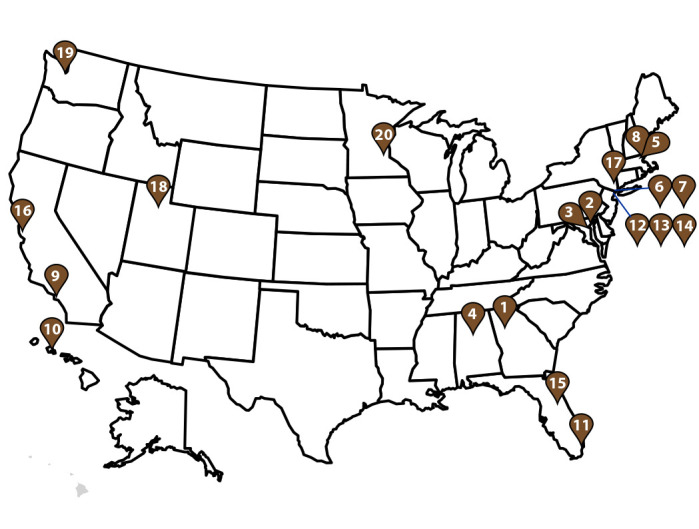
U.S. GeoSentinel sites* — GeoSentinel Network, 2012–2021 * Sites include Atlanta, GA (1); Baltimore, MD (2); Bethesda, MD (3); Birmingham, AL (4); Boston, MA (5); Bronx, NY (6); Bronx Lebanon, NY (7); Cambridge, MA (8); Hollywood, CA (9); Honolulu, HI (10); Miami, FL (11); New York City, NY (12); New York Northwest, NY (13); New York West, NY (14); Orlando, FL (15); Palo Alto, CA (16); Peekskill, NY (17); Salt Lake City, UT (18); Seattle, WA (19); and St. Paul, MN (20).

This report includes GeoSentinel data limited to unique patients with ≥1 confirmed or probable travel-related diagnosis who were evaluated after migration or travel at a GeoSentinel site in the United States during 2012–2021. Each patient might have multiple diagnoses. Patients must have been residents of the United States and evaluated after travel and within 10 years of migrating or returning from a trip outside of the United States. Only migrants with illnesses associated with their migration to the United States were included. The validity of diagnoses was verified by an infectious disease specialist using the diagnostic methods recorded by the sites. Descriptive analyses were performed on data from the 20 GeoSentinel sites in the United States (Figure 2) with patients who met inclusion criteria. Frequencies were calculated on patient demographics (e.g., sex, age, country of birth, citizenship, and residence), travel-related information (e.g., reason for travel and country or region of exposure), diagnosis, diagnostic methods, year of illness onset, and severity of illness. Because of changes in the collection of travel-related information, a subanalysis was done on travel-related information before and after November 16, 2018. This information is reported separately. Geographic regions of exposure are classified based on modified UNICEF groupings (https://data.unicef.org/regionalclassifications/). Data were managed using Microsoft Access (version 2208; Microsoft Corporation), and all analyses were performed using SAS (version 9.4; SAS Institute).

## Selected Worldwide Health Event Notifications

To demonstrate GeoSentinel’s ability to identify sentinel events and emerging disease patterns, three examples (i.e., dengue in Angola [2013], Zika in Costa Rica [2016], and yellow fever in Brazil [2018]) of emerging sentinel health threats that occurred during 2012–2021 are described. These health events were not limited to residents of the United States who were evaluated after travel and ≤10 years of migrating or returning from a trip outside of the United States. Therefore, these patients could be residents of any country and were seen at GeoSentinel sites both inside and outside of the United States.

## Results

**FIGURE 3:**
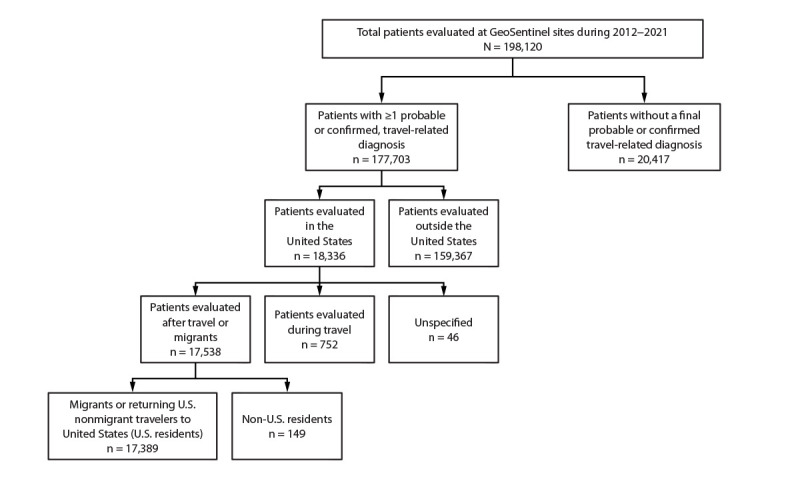
U.S. nonmigrant travelers or migrants presenting to U.S. GeoSentinel sites — GeoSentinel Network, 2012–2021* * A total of 149 non-U.S. residents were excluded from the analysis.

During 2012–2021, a total of 198,120 unique patients were evaluated at GeoSentinel sites globally and included in GeoSentinel’s database (Figure 3). Of these, 177,703 patients received at least one confirmed or probable travel-related diagnosis, of which 18,336 were reported from 20 GeoSentinel sites in the United States. Of the 17,538 patients evaluated by a clinician after travel, 17,389 were migrants or returning U.S. nonmigrant travelers to the United States, accounting for 25,973 travel-related diagnoses. The remaining 149 patients were non-U.S. residents and were excluded from the analysis. The results of migrants and returning nonmigrant travelers are reported separately.

### Migrants

#### Patient Demographics

Of the 17,389 patients who were included in this analysis, 7,530 (43.3%) were recent migrants to the United States; <1% of patients were expatriates. Of 7,527 migrants, 47.4% were female (Table 1). The median age was 28.5 years (range = <19 years to 93 years), and the largest proportion of migrants was aged 19–39 years (35.9%). Of 4,672 patients with information available, 88.8% did not receive pretravel health information. Of 2,867 patients with information available on severity, a majority (89.8%) were seen as outpatients, 9.7% were seen in an inpatient ward, and <1% were seen in an ICU.

**TABLE 1:** Number of migrants and nonmigrant travelers with at least one confirmed or probable travel-related diagnosis, by selected characteristics — GeoSentinel Network, United States, 2012–2021

Characteristic	Migrants N = 13,986 No. (%)	Nonmigrant travelers N = 11,987 No. (%)
Sex*		
Female	3,565 (47.4)	5,492 (55.7)
Male	3,962 (52.6)	4,360 (44.3)
**Age, yrs^†^**		
<19	2,485 (33.2)	933 (9.5)
19–39	2,691 (35.9)	4,323 (44.1)
40–59	1,550 (20.7)	2,917 (29.8)
≥60	764 (10.2)	1,620 (16.5)
**Place of birth, citizenship, and residence**		
Born in the United States	0 (—)	7,425 (75.3)
Citizen of the United States^§^	448 (6.0)	8,799 (89.4)
Resident of the United States	7,530 (100)	9,859 (100)
**Pretravel encounter^¶^**		
Yes	524 (11.2)	3,089 (34.4)
No	4,148 (88.8)	5,878 (65.6)
**Expatriate**		
Yes	21 (0.3)	111 (1.1)
No	7,509 (99.7)	9,748 (98.9)
**Year**		
2012	1,189 (15.8)	1,516 (15.4)
2013	1,057 (14.0)	1,271 (12.9)
2014	1,032 (13.7)	1,204 (12.2)
2015	968 (12.9)	1,166 (11.8)
2016	933 (12.4)	1,112 (11.3)
2017	930 (12.4)	1,067 (10.8)
2018	489 (6.5)	971 (9.9)
2019	421 (5.6)	951 (9.7)
2020	282 (3.8)	383 (3.9)
2021	229 (3.0)	218 (2.2)

* Information available for 7,527 migrants and 9,852 other travelers.

^†^ Information available for 7,490 migrants and 9,793 other travelers.

^§^ Information available for 7,488 migrants and 9,841 other travelers.

^¶^ Information available for 4,672 migrants and 8,967 other travelers.

#### Diagnoses

Of the 13,986 travel-related diagnoses among migrants, the most frequent were vitamin D deficiency (20.2%), *Blastocystis* (10.9%), latent tuberculosis (10.3%), strongyloidiasis (6.7%), and eosinophilia (5.8%) (Table 2). A total of 43% of diagnoses fell into eight infectious or travel-related syndrome groupings including “other” (18.7%), gastrointestinal (15.7%), dermatological (2.0%), neurologic (1.9%), genitourinary (1.6%), febrile (1.5%), respiratory (1.5%), and musculoskeletal (<1%). No deaths or animal bites or scratches were reported (Table 3).

**TABLE 2:** Number of most common confirmed and probable travel-related diagnoses in migrants and nonmigrant travelers — GeoSentinel Network, United States, 2012–2021

Migrants N = 13,986		Nonmigrant travelers N = 11,987	
Diagnosis	No. (%)	Diagnosis	No. (%)
Vitamin D deficiency	2,820 (20.2)	Diarrhea, acute	2,031 (16.9)
*Blastocystis*	1,527 (10.9)	Viral syndrome	581 (4.9)
Latent tuberculosis	1,444 (10.3)	Irritable bowel syndrome	493 (4.1)
Strongyloidiasis	932 (6.7)	Malaria	421 (3.5)
Eosinophilia	804 (5.8)	Campylobacteriosis	371 (3.1)
Anemia	598 (4.3)	Insect or other arthropod bite or sting	307 (2.6)
Dental problem	470 (3.4)	Giardiasis	284 (2.4)
Giardiasis	417 (3.0)	Dengue	275 (2.3)
Abnormal urinalysis	397 (2.8)	Diarrhea, chronic	271 (2.3)
Other nonpathogenic protozoa	369 (2.6)	Influenza-like illness	256 (2.1)

**TABLE 3:** Number of travel-related diagnoses* of migrants and nonmigrant travelers in syndrome and system groupings — GeoSentinel Network, United States, 2012–2021^†^

Migrants N = 13,986		Nonmigrant travelers N = 11,987	
Diagnosis	No. (%)	Diagnosis	No. (%)
**Other**	**2,614 (18.7)**	**Gastrointestinal**	**5,173 (43.2)**
Latent tuberculosis	1,444 (55.2)	Diarrhea, acute	2,031 (39.3)
Eosinophilia	804 (30.8)	Irritable bowel syndrome (new onset)	493 (9.5)
Chagas disease	100 (3.8)	Campylobacteriosis	371 (7.2)
Posttraumatic stress disorder	94 (3.6)	Giardiasis	284 (5.5)
Depression	75 (2.9)	Diarrhea, chronic	271 (5.2)
**Gastrointestinal**	**2,202 (15.7)**	**Febrile**	**2,001 (16.7)**
Strongyloidiasis, simple intestinal	917 (41.6)	Viral syndrome, with or without rash	581 (29.0)
Giardiasis	417 (18.9)	Malaria	421 (21.0)
*Helicobacter pylori* infection	186 (8.4)	Dengue	275 (13.7)
Dientamoebiasis	154 (6.9)	Chikungunya	128 (6.4)
Schistosomiasis	143 (6.5)	Febrile illness, unspecified	100 (5.0)
**Dermatological**	**275 (2.0)**	**Respiratory or ENT**	**1,554 (13.0)**
Fungal infection	117 (42.6)	Influenza-like illness	256 (16.5)
Insect bite or sting	30 (10.9)	Respiratory tract infection, upper	231 (14.9)
Rash, unknown etiology	28 (10.2)	Bronchitis, acute	185 (11.9)
Leishmaniasis, cutaneous	16 (5.8)	Sinusitis, acute	142 (9.1)
Leprosy	12 (4.4)	Pneumonia, lobar unspecified	130 (8.4)
**Neurologic**	**263 (1.9)**	**Dermatological**	**1,071 (8.9)**
Neurocysticercosis	202 (76.8)	Insect or arthropod bite or sting	335 (31.3)
Headache	43 (16.4)	Rash, unknown etiology	92 (8.8)
Ataxia	4 (1.5)	Rash, dermatitis	82 (7.9)
Tuberculosis, CNS tuberculoma	4 (1.5)	Skin and soft tissue infection	77 (7.4)
Tuberculosis, meningitis	3 (1.1)	Skin and soft tissue infection, superficial	63 (6.0)
**Genitourinary and STDs**	**229 (1.6)**	**Other**	**487 (4.1)**
Schistosomiasis	63 (27.5)	Dehydration	91 (18.7)
Chlamydia, genital	35 (15.3)	Jet lag	87 (17.9)
Syphilis	26 (11.4)	Eosinophilia	57 (11.7)
Urinary tract infection	25 (10.9)	Latent tuberculosis	43 (8.8)
HIV, newly diagnosed	23 (10.0)	Anxiety disorder	37 (7.6)
**Febrile**	**212 (1.5)**	**Genitourinary and STDs**	**173 (1.4)**
Malaria	54 (25.5)	Urinary tract infection	57 (33.0)
Tuberculosis, other extrapulmonary	28 (13.2)	Schistosomiasis	20 (11.6)
Toxoplasmosis	17 (8.0)	Gonorrhea	16 (9.3)
Tuberculosis, lymphadenitis	14 (6.6)	Pyelonephritis	15 (8.7)
Tuberculosis, disseminated	11 (5.2)	Chlamydia, genital	14 (8.1)
**Respiratory or ENT**	**204 (1.5)**	**Animal bites or scratches**	**153 (1.3)**
Tuberculosis, pulmonary	144 (70.6)	Dog bite	77 (50.3)
Otitis media, acute	10 (4.9)	Monkey bite	28 (18.3)
Pneumonia, atypical	7 (3.4)	Other animal bite	10 (6.5)
Otitis externa	6 (2.9)	Monkey exposure	9 (5.9)
Pneumonia, lobar, unspecified	6 (2.9)	Dog exposure	6 (3.9)
**Musculoskeletal**	**131 (<1.0)**	**Musculoskeletal**	**142 (1.2)**
Arthralgia	63 (48.1)	Arthralgia	28 (19.7)
Trauma or injury	57 (43.5)	Fracture	25 (17.6)
Osteomyelitis	2 (1.5)	Myalgia	15 (10.6)
Knee pain	2 (1.5)	Trauma or injury	14 (9.9)
Sprain	2 (1.5)	Contusion	11 (7.8)
**Animal bites or scratches**	0 (—)	**Neurologic**	**105 (<1)**
None	0 (—)	Headache	28 (26.7)
		Vertigo	13 (12.4)
		Acute mountain sickness	11 (10.5)
		Neurocysticercosis	10 (9.5)
		Dizziness	9 (8.6)

**Abbreviations:** CNS = central nervous system; ENT = ear, nose, and throat; STD = sexually transmitted disease.

* The five most common diagnoses are provided for the most common travel-related syndrome and system groupings.

^†^ No deaths were observed among migrants; four deaths were observed among nonmigrant travelers.

Of the 2,614 diagnoses in the “other” grouping (Table 3), the most frequent were latent tuberculosis (55.2%), eosinophilia (30.8%), Chagas disease (3.8%), posttraumatic stress disorder (3.6%), and depression (2.9%). Of the 2,202 diagnoses in the gastrointestinal grouping, the most frequent were simple intestinal strongyloidiasis (41.6%), giardiasis (18.9%), *Helicobacter pylori* infection (8.4%), dientamoebiasis (6.9%), and schistosomiasis (6.5%). Of the 275 diagnoses in the dermatological grouping, the most frequent were fungal infection (42.6%), insect bite/sting (10.9%), rash of unknown etiology (10.2%), cutaneous leishmaniasis (5.8%), and leprosy (4.4%). Of the 263 diagnoses in the neurologic grouping, the most frequent were neurocysticercosis (76.8%), headache (16.4%), ataxia (1.5%), central nervous system tuberculosis (1.5%), and tuberculosis meningitis (1.1%). Of the 229 diagnoses in the genitourinary grouping, the most frequent were schistosomiasis (27.5%), chlamydia (15.3%), syphilis (11.4%), urinary tract infection (10.9%), and HIV (10.0%).

Among the 212 diagnoses in the febrile grouping (Table 3), the most frequent were malaria (25.5%), other extrapulmonary tuberculosis (13.2%), toxoplasmosis (8.0%), tuberculosis lymphadenitis (6.6%), and disseminated tuberculosis (5.2%). Malaria was diagnosed in 54 (<1%) migrants, and 88.5% did not receive pretravel health information (information available for 26 migrants). Of all species of malaria, *Plasmodium falciparum* was diagnosed most frequently (77.4%).

Among the 204 diagnoses in the respiratory grouping (Table 3), the most frequent was pulmonary tuberculosis (70.6%), which accounted for 68.9% of all active tuberculosis diagnoses; only 1% of migrants received a diagnosis of active tuberculosis disease. The remaining frequent diagnoses in the respiratory grouping were acute otitis media (4.9%), atypical pneumonia (3.4%), otitis externa (2.9%), and unspecified lobar pneumonia (2.9%). Of the 131 diagnoses in the musculoskeletal grouping, the most frequent were arthralgia (48.1%), trauma or injury (43.5%), osteomyelitis (1.5%), knee pain (1.5%), and sprain (1.5%).

##### Diagnostic Characteristics Before November 16, 2018

Among the 2,892 diagnoses with information available (Table 4), the five most frequent regions of exposure were Sub-Saharan Africa (22.7%), the Caribbean (21.3%), Central America (13.4%), South East Asia (13.1%), and South Central Asia (9.2%). Among the 2,554 diagnoses with information available, the most frequent countries of exposure were Dominican Republic (7.9%), Thailand (6.5%), Haiti (6.2%), Ecuador (4.8%), and Myanmar (4.3%). Of 46 migrants with a malaria diagnosis, 89.3% were exposed in Sub-Saharan Africa (information available for 28 migrants).

**TABLE 4:** Number of diagnoses among migrants, by region or country of exposure — GeoSentinel Network, United States, 2012–2021

Before November 16, 2018 N = 11,974		After November 16, 2018 N = 2,012	
Site	No. (%)	Site	No. (%)
Exposure region*^,†^		**Exposure region^†^**	
Sub-Saharan Africa	655 (22.7)	Central America	556 (27.6)
Caribbean	615 (21.3)	Sub-Saharan Africa	528 (26.2)
Central America	387 (13.4)	South East Asia	340 (16.9)
South East Asia	379 (13.1)	Caribbean	169 (8.4)
South Central Asia	265 (9.2)	South America	141 (7.0)
**Exposure country^¶,§^**		**Exposure country^†,¶^**	
Dominican Republic	201 (7.9)	El Salvador	177 (11.2)
Thailand	165 (6.5)	Thailand	169 (10.7)
Haiti	157 (6.2)	Honduras	143 (9.1)
Ecuador	122 (4.8)	Guatemala	120 (7.6)
Myanmar	109 (4.3)	Dominican Republic	93 (5.9)

* Information available for 2,892 diagnoses.

^†^ Five countries or regions with highest number of patient exposures.

^§^ Information available for 2,554 diagnoses.

^¶^ Information available for 1,575 diagnoses.

##### Diagnostic Characteristics After November 16, 2018

Among the 2,012 diagnoses with information available (Table 4), the five most frequent regions of exposure were Central America (27.6%), Sub-Saharan Africa (26.2%), South East Asia (16.9%), the Caribbean (8.4%), and South America (7.0%). Among the 1,575 diagnoses with information available, the most frequent countries of exposure were El Salvador (11.2%), Thailand (10.7%), Honduras (9.1%), Guatemala (7.6%), and Dominican Republic (5.9%). Of seven migrants with a malaria diagnosis, all were exposed in Sub-Saharan Africa (information available for seven migrants).

### Returning Nonmigrant Travelers

#### Patient Demographics

Among the 9,859 nonmigrant travelers returning to the United States, 55.7% were female and 75.3% were born in the United States. The median age was 37 years (range = <19 years to 96 years), and the largest proportion of nonmigrant travelers was aged 19–39 years (44.1%). Among the 8,967 patients with information available, 65.6% did not receive pretravel health information. Among the 5,884 patients with information available on severity, a majority (90.6%) were seen as outpatients, 8.4% were seen in an inpatient ward, and <1% were seen in an ICU. Approximately 1% of patients were expatriates, and 89.4% were U.S. citizens.

#### Diagnoses

Of the 11,987 travel-related diagnoses of returning U.S. nonmigrant travelers (Table 3), 90.7% of diagnoses fell into nine infectious or travel-related syndrome groupings, including gastrointestinal (43.2%), febrile (16.7%), respiratory (13.0%), dermatological (8.9%), “other” (4.1%), animal bites or scratches (1.3%), genitourinary (1.4%), musculoskeletal (1.2%), and neurologic (<1%). The most frequent diagnoses (Table 2) were acute diarrhea (16.9%), viral syndrome (4.9%), irritable bowel syndrome (4.1%), campylobacteriosis (3.1%), and malaria (3.5%). Four deaths were reported, of which two were patients who received a diagnosis of severe *P. falciparum* malaria. Of the remaining two patients, one received a diagnosis of COVID-19 and the other received a diagnosis of acute unspecified hepatitis with renal failure.

Among the 5,173 diagnoses in the gastrointestinal grouping (Table 3), the most frequent were acute diarrhea (39.3%), irritable bowel syndrome (9.5%), campylobacteriosis (7.2%), giardiasis (5.5%), and chronic diarrhea (5.2%). Among the 2,001 diagnoses in the febrile grouping, the most frequent were viral syndrome (29.0%), malaria (21.0%), dengue (13.7%), chikungunya (6.4%), and unspecified febrile illness (5.0%). Among the 421 nonmigrant travelers with malaria of any species diagnosed, 80.8% had *P. falciparum*.

Among the 1,554 diagnoses in the respiratory grouping (Table 3), the most frequent were influenza-like illness (16.5%), upper respiratory tract infection (14.9%), acute bronchitis (11.9%), acute sinusitis (9.1%), and unspecified lobar pneumonia (8.4%). Among the 1,071 diagnoses in the dermatological grouping, the most frequent were insect or arthropod bite or sting (31.3%), rash of unknown etiology (8.8%), dermatitis (7.9%), skin and soft tissue infection (e.g., erysipelas, cellulitis, or gangrene [7.4%]), and superficial skin and soft tissue infection (6.0%). Among the 487 diagnoses in the “other” grouping, the most frequent were dehydration (18.7%), jet lag (17.9%), eosinophilia (11.7%), latent tuberculosis (8.8%), and anxiety disorder (7.6%).

Among the 173 diagnoses in the genitourinary grouping (Table 3), the most frequent were urinary tract infection (33.0%), schistosomiasis (11.6%), gonorrhea (9.3%), pyelonephritis (8.7%), and genital chlamydia (8.1%). Among the 142 diagnoses in the musculoskeletal grouping, the most frequent were arthralgia (19.7%), fracture (17.6%), myalgia (10.6%), trauma or injury (9.9%), and contusion (7.8%). Among the 105 diagnoses in the neurologic grouping, the most frequent were headache (26.7%), vertigo (12.4%), acute mountain sickness (10.5%), neurocysticercosis (9.5%), and dizziness (8.6%). Among the 153 diagnoses of bites or scratches, the most frequent were dog bite (50.3%), monkey bite (18.3%), other animal bite (6.5%), monkey exposure (5.9%), and dog exposure (3.9%).

##### Diagnostic Characteristics Before November 16, 2018

Among the 9,919 diagnoses, 6,518 had information regarding travel reason (Table 5). The most frequent reasons for travel were tourism (44.8%), VFR (22.0%), and business (13.4%). Among the 6,296 diagnoses with information available, the five most frequent regions of exposure were Central America (19.2%), Sub-Saharan Africa (17.7%), the Caribbean (13.0%), South East Asia (10.4%), and South America (9.4%). Among 5,920 diagnoses with information available, the most frequent countries of exposure were Mexico (12.5%), India (7.2%), Dominican Republic (5.3%), China (3.3%), and Costa Rica (3.0%).

**TABLE 5:** Number of diagnoses among nonmigrant travelers, by travel reason and region or country of exposure — GeoSentinel Network, United States, 2012–2021

Before November 16, 2018 N = 9,919		After November 16, 2018 N = 2,068	
Site	No. (%)	Site	No. (%)
Travel reason*		**Travel reason**	
Tourism	2,919 (44.8)	Tourism	1,109 (53.6)
Visiting friends and relatives	1,432 (22.0)	Visiting friends and relatives	443 (21.4)
Business	871 (13.4)	Business	254 (12.3)
Missionary	852 (13.1)	Missionary	129 (6.2)
Student	343 (5.3)	Student	92 (4.4)
Research	63 (1.0)	Providing medical care	18 (<1)
Military	17 (<1)	Not ascertainable	8 (<1)
Planned medical care	16 (<1)	Planned medical care	8 (<1)
Migrant worker	4 (<1)	Military	4 (<1)
Providing medical care	1 (<1)	Retirement	2 (<1)
		Migrant worker	1 (<1)
**Exposure region^†,§^**		**Exposure region^§^**	
Central America	1,210 (19.2)	Sub-Saharan Africa	528 (25.5)
Sub-Saharan Africa	1,114 (17.7)	Central America	357 (17.3)
Caribbean	821 (13.0)	South East Asia	232 (11.2)
South East Asia	654 (10.4)	Caribbean	225 (10.9)
South America	594 (9.4)	South Central Asia	186 (9.0)
**Exposure country^§,¶^**		Exposure country^§,^**	
Mexico	738 (12.5)	Mexico	249 (13.2)
India	426 (7.2)	India	95 (5.0)
Dominican Republic	312 (5.3)	Dominican Republic	80 (4.2)
China	198 (3.3)	Philippines	57 (3.0)
Costa Rica	179 (3.0)	Ethiopia	56 (3.0)

* Information available for 6,518 diagnoses.

^†^ Information available for 6,296 diagnoses.

^§^ Five countries or regions with highest number of patient exposures.

^¶^ Information available for 5,920 diagnoses.

** Information available for 1,894 diagnoses.

Of 300 nonmigrant travelers with malaria, 70.3% were VFRs (information available for 232 nonmigrant travelers), and 88.6% were exposed in Sub-Saharan Africa. Of 163 VFRs with malaria, 70.2% did not receive pretravel health information (information available for 141 nonmigrant travelers), and 88.3% did not take malaria chemoprophylaxis (information available for 103 nonmigrant travelers).

##### Diagnostic Characteristics After November 16, 2018

Information regarding travel reason and exposure region was available for all 2,068 diagnoses (Table 5). The most frequent reasons for travel were tourism (53.6%), VFR (21.4%), business (12.3%), and missionary (6.2%). The five most frequent regions of exposure were Sub-Saharan Africa (25.5%), Central America (17.3%), South East Asia (11.2%), the Caribbean (10.9%), and South Central Asia (9.0%). Among the 1,894 diagnoses with information available, the most frequent countries of exposure were Mexico (13.2%), India (5.0%), Dominican Republic (4.2%), Philippines (3.0%), and Ethiopia (3.0%).

Of 121 nonmigrant travelers with malaria, 57.9% were VFRs and 95.9% were exposed in Sub-Saharan Africa. Of 70 VFRs with malaria, 83.3% did not receive pretravel health information (information available for 54 nonmigrant travelers), and none took malaria chemoprophylaxis (information available for three nonmigrant travelers).

## Selected Health Event Notifications in GeoSentinel

### Dengue in Angola, 2013

During April–May 2013, GeoSentinel sites in Canada, France, Germany, Israel, and South Africa reported 10 cases of dengue among travelers returning from Luanda, Angola. All patients had classic symptoms of dengue that included headache and joint pain and recovered without complication. Although dengue is endemic in Angola, before 2013, the last outbreak occurred during the 1980s. In the decades that followed, little was known regarding the epidemiology of dengue in Angola because of poor surveillance (*17*).

Although six cases of dengue had been reported to the Ministry of Health of Angola by April 1, 2013, the GeoSentinel cases, in combination with other imported cases to Portugal, were among the first indications of a large-scale outbreak. By May 31, there were 517 suspected cases and one death reported; all but two cases were in Luanda province (*18*). The GeoSentinel cases in Angola demonstrated that data on travelers’ adverse health events can aid in the detection of outbreaks, offering insight into the epidemiology of infectious disease in countries with suboptimal surveillance and reporting.

### Zika in Costa Rica, 2016

On January 26, 2016, a GeoSentinel site in Massachusetts diagnosed dengue in a returned U.S. traveler from Nosara, Costa Rica. The patient returned to the United States with fever, rash, conjunctivitis, arthralgia, and headache; the patient also reported multiple mosquito bites. The patient was referred to a GeoSentinel site where antibody tests for Zika and dengue viruses were conducted by CDC. Plaque reduction neutralization antibody testing confirmed a diagnosis of Zika (*19*).

Zika virus emerged in the western hemisphere during 2014–2016 when outbreaks were reported from certain countries in the Americas and Caribbean (*20*). This case was the first case of Zika reported from Costa Rica, illustrating the continual geographic spread of a high-consequence pathogen. The Massachusetts GeoSentinel site detected and reported this sentinel case, and it also sent a networkwide notification, alerting clinicians to the risk for Zika in Costa Rica, a popular travel destination with no previous evidence of underlying circulation. By August 2017, a total of 1,920 cases were reported in Costa Rica, mirroring the trends of other countries in the region.

### Yellow Fever in Brazil, 2018

In January 2018, a GeoSentinel site in the Netherlands reported a case of yellow fever in a Dutch man aged 46 years with recent travel to São Paulo state, Brazil. He had signs and symptoms of diarrhea, fever, headache, myalgia, and vomiting. By March 15, 2018, four additional GeoSentinel sites reported cases of yellow fever among travelers returning from Brazil, including two deaths. These five cases accounted for one half of all cases reported among international travelers to Brazil during this time. All patients were unvaccinated travelers, many of whom visited Ilha Grande (*21*).

Although yellow fever is endemic in Brazil, during 2016–2017 and 2017–2018, a higher incidence does not, by itself, indicate geographic expansion (*22*). Cases detected by GeoSentinel in early 2018 were among the first reported in newly identified regions of risk, confirming travelers as sentinels in the expansion of the outbreak and highlighting the importance of yellow fever vaccination in recommended regions (*21*).

## Discussion

GeoSentinel is the only surveillance network that operates a global, provider-based emerging infections sentinel network to conduct surveillance for travel-related infections and communicates with public health and clinical partners (*11*). From its inception in 1995 to 2011 (*11*), efforts were made to increase the size of the network, modernize data collection, and introduce internal validation to improve the quality of the data collected. Since 2012, GeoSentinel has expanded to 71 sites on six continents and has generated approximately 70 peer-reviewed publications. GeoSentinel also has undergone numerous methodologic changes aimed to improve data collection, the validity of resulting conclusions, and the provision of public health recommendations.

GeoSentinel data have been instrumental in the detection of sentinel events, as demonstrated by, but not limited to, the detection of expanded geographic area of yellow fever in Brazil (*21*), a large outbreak of dengue in Angola (*17*), and the first case of Zika in Costa Rica (*19*). These examples illustrate GeoSentinel’s ability to both identify emerging pathogens and communicate findings with clinicians and public health professionals around the world.

The most frequent diagnoses among migrants described in this analysis (e.g., vitamin D deficiency and latent tuberculosis) have been described elsewhere (*23*,*24*). Vitamin D deficiency might be because of reduced sun exposure caused by skin-covering clothing as well as low dietary intake (*23*). Acquisition of strongyloidiasis and latent tuberculosis might be from crowding, malnutrition, exposure to unsafe food and water, inadequate sanitation, and limited access to health care (*25*). Multiple presentations of *Mycobacterium tuberculosis* (e.g., pulmonary, extrapulmonary, lymphadenitis, disseminated, CNS tuberculoma, and meningitis) also were reported among migrants, highlighting that health care professionals should maintain a high degree of suspicion for *M. tuberculosis* infection among ill patients whose routine bacterial cultures do not yield a pathogen. Because the United States has the largest population of migrants in the world (*26*), health care professionals should continue to advocate for medical care for this underserved population, with the aim to prevent disease reactivation and subsequent spread to and within vulnerable populations.

Gastrointestinal illnesses remain a frequent cause of illness among travelers (*27*). In this analysis, acute diarrhea was the most frequent illness among nonmigrant travelers, accounting for 16.9% of their diagnoses. Previous studies have reported attack rates for acute diarrhea among travelers ranging from 30%–70%, most often caused by bacterial pathogens and transmitted because of poor hygiene practices in local restaurants (*28*). In other studies, travelers have reported not adhering to prevention practices and drinking unsafe tap water, consuming drinks with ice, eating salads, and consuming unpasteurized dairy products while abroad (*29*). Most acute diarrhea cases reported to GeoSentinel were of unknown etiology, illustrating the lack of use of specialized diagnostic tests or culture to determine the cause of diarrhea (*30*), despite the widespread availability of multiplex polymerase chain reaction tests for gastrointestinal pathogens (*31*), likely because the majority of cases of acute diarrhea resolve without the need for intervention (*32*).

Febrile illnesses were another frequent cause of illness among nonmigrant travelers in this analysis, of which viral syndromes and *P. falciparum* malaria were most frequent. Of nonmigrant travelers with malaria, a majority were exposed in Sub-Saharan Africa; the majority were VFRs, who infrequently received pretravel health advice or took malaria chemoprophylaxis. Inadequate pretravel preparation practices place VFRs at high risk for acquiring malaria during travel. Studies of African VFR travelers indicated they might not be able to afford health care visits, might feel unable to advocate for themselves in a health care setting, and might be culturally opposed to malaria chemoprophylaxis or other preventive measures (e.g., use of bed nets) because of concerns about offending their hosts or a low perception of risk (*33*,*34*). CDC recommends that all travelers going to an area where malaria is endemic take chemoprophylaxis before and during travel (*35*), but special considerations (e.g., improving accessibility or improving trust in the U.S. health care system) could be prioritized to ensure that VFRs are protected from malaria (*33*,*36*).

### COVID-19 Pandemic

During 2020–2021, the number of patients presenting at U.S. GeoSentinel sites substantially decreased, mirroring worldwide declines in travel because of the COVID-19 pandemic and associated travel restrictions. Although GeoSentinel historically has been lauded for its ability to detect sentinel events in real time, GeoSentinel only retrospectively identified cases of influenza-like illness as COVID-19 among travelers who returned from China early in the pandemic. Although the outbreak began in China, a popular destination for U.S. travelers, in late 2019, U.S. GeoSentinel sites first reported COVID cases among travelers in March 2020. This lack of early identification of COVID-19 cases was likely because of three main reasons. First, daily reports were generated for assistance in detecting sentinel events, but these were simple line listings of cases and focused primarily on etiologic diagnoses; although cases of “viral illness” were reported from China to GeoSentinel as early as December 2019, these were not identified to be out of the ordinary. Surveillance systems (e.g., GeoSentinel) are most effective in detecting established etiologic illnesses, not novel pathogens (*37*). Second, delays in identification and available diagnostics for this novel pathogen meant that testing was not routinely available globally or at GeoSentinel sites early in the pandemic; therefore, etiologic COVID-19 diagnoses were only made retrospectively. Third, many cases of COVID-19 might have had mild symptoms similar to influenza, the common cold, and seasonal allergies, whose symptoms can be treated with over-the-counter medication. Thus, ill travelers might have opted to treat their symptoms at home and not seek health care or visited their primary care provider instead of a travel and tropical medicine site despite their recent travel.

To address these challenges, GeoSentinel has begun to explore other ways to detect and track novel pathogens more rapidly. GeoSentinel is developing automated, real-time data analytics (e.g., machine learning algorithms by likelihood of outbreak origin) to improve the ability to detect outbreaks and unusual clusters of disease together with more classical surveillance approaches (*38*).

## Limitations

The findings in this report are subject to at least six limitations. First, GeoSentinel data are not representative of all travelers. Although GeoSentinel tracks illnesses among travelers who are treated at GeoSentinel sites, data are entered at the discretion of the sites, which might lead to underreporting. Second, sites are not evenly dispersed globally and are predominantly located in Europe and North America. This pattern might reflect the travel attributes of persons from these continents. Third, GeoSentinel only collects data on ill travelers who seek care at GeoSentinel sites. The total numbers of travelers, ill travelers who do not seek care, or travelers who seek care outside of the GeoSentinel network is unknown. Thus, GeoSentinel data cannot be used to estimate risk, incidence, prevalence, or other rates because the number of well or unexposed travelers in the denominator is not known. Fourth, the United States does not have many large travel and tropical medicine centers (in comparison with Europe or Asia) and travelers, including migrants, might seek care external to the GeoSentinel network. Fifth, although changes in the information collected, methods, and the sites themselves have made data collection more robust, these changes also make the comparison of periods difficult and, in certain cases, inappropriate. Although all sites use the same standardized data collection form, data entry practices vary by site and over time. Finally, the large number of migrants reported from U.S. GeoSentinel sites might be the result of selection bias because of the migration medicine specialization of many U.S. sites. Diseases detected among migrants might be driven by routine screening on entry to the United States.

## Future Directions

As of September 2021, GeoSentinel has incorporated research through its cooperative agreement between CDC and ISTM. This will allow GeoSentinel to conduct hypothesis-driven studies to help guide clinical and public health recommendations. Initial projects include investigation of fever of unknown etiology among travelers, neurocognitive outcomes among travelers with malaria, kinetics of human Mpox infections, and exploration of the distribution and types of antimalarial resistance using malaria genomics.

## Conclusion

Over the past decade, GeoSentinel has contributed to the early detection of diseases among international travelers. The information about demographics, traveler types, and frequent diagnoses provides data that clinicians and public health agencies can use to improve pretravel preparedness and enhance guidance for the evaluation and treatment of ill travelers who seek medical care after international travel. The key successes and shortcomings of GeoSentinel serve as references to improve surveillance and expand the capability to detect sentinel events.

## Footnotes

* 45 C.F.R. part 46.102(l)(2), 21 C.F.R. part 56; 42 U.S.C. §241(d); 5 U.S.C. §552a; 44 U.S.C. §3501 et seq.

## References

[1] Glaesser D, Kester J, Paulose H, Alizadeh A, Valentin B. Global travel patterns: an overview. J Travel Med 2017;24:1–5. 10.1093/jtm/tax007 28637267

[2] World Tourism Organization. Yearbook of tourism statistics, data 2015–2019, 2021 edn. Madrid, Spain: World Tourism Organization; 2021. https://www.e-unwto.org/doi/book/10.18111/9789284422487

[3] Angelo KM, Kozarsky PE, Ryan ET, Chen LH, Sotir MJ. What proportion of international travellers acquire a travel-related illness? a review of the literature. J Travel Med 2017;24:1–8. 10.1093/jtm/tax046 28931136 PMC5825178

[4] Kozarsky PE, Keystone JS. VFR (visiting friends and relatives) travelers [Chapter 5]. In: Schwartz E, ed. Tropical diseases in travelers. Chichester, UK: Wiley-Blackwell; 2010: 35–44.

[5] Ghent A. Overcoming migrants’ barriers to health. Bull World Health Organ 2008;86:583–4. 10.2471/BLT.08.020808 18797611 PMC2649480

[6] Findlater A, Bogoch II. Human mobility and the global spread of infectious diseases: a focus on air travel. Trends Parasitol 2018;34:772–83. 10.1016/j.pt.2018.07.004 30049602 PMC7106444

[7] Angelo KM, Gastañaduy PA, Walker AT, Spread of measles in Europe and implications for US travelers. Pediatrics 2019;144:e20190414. 10.1542/peds.2019-0414 31209161 PMC6657509

[8] CDC, Guinea Interministerial Committee for Response Against the Ebola Virus, World Health Organization, Liberia Ministry of Health and Social Welfare, Sierra Leone Ministry of Health and Sanitation. Update: Ebola virus disease epidemic—West Africa, February 2015. MMWR Morb Mortal Wkly Rep 2015;64:186–7. 25719681 PMC5779600

[9] Keni R, Alexander A, Nayak PG, Mudgal J, Nandakumar K. COVID-19: emergence, spread, possible treatments, and global burden. Front Public Health 2020;8:216. 10.3389/fpubh.2020.00216 32574299 PMC7270802

[10] Chen LH, Wilson ME. The role of the traveler in emerging infections and magnitude of travel. Med Clin North Am 2008;92:1409–32, xi. 10.1016/j.mcna.2008.07.005 19061759 PMC7094659

[11] Harvey K, Esposito DH, Han P, ; CDC. Surveillance for travel-related disease—GeoSentinel Surveillance System, United States, 1997–2011. MMWR Surveill Summ 2013;62(No. SS-3):1–23. 23863769

[12] Hamer DH, Rizwan A, Freedman DO, Kozarsky P, Libman M. GeoSentinel: past, present and future. Alpharetta, GA: International Society of Travel Medicine, Journal of Travel Medicine; 2020. https://academic.oup.com/jtm/article/27/8/taaa219/6007543 10.1093/jtm/taaa219 PMC7799014 33247586

[13] Gautret P, Angelo KM, Asgeirsson H, ; GeoSentinel Network. International mass gatherings and travel-associated illness: a GeoSentinel cross-sectional, observational study. Travel Med Infect Dis 2019;32:101504. 10.1016/j.tmaid.2019.101504 31707112 PMC7110217

[14] Gautret P, Angelo KM, Asgeirsson H, ; GeoSentinel Global Surveillance Network. Rabies post-exposure prophylaxis started during or after travel: a GeoSentinel analysis. PLoS Negl Trop Dis 2018;12:e0006951. 10.1371/journal.pntd.0006951 30422981 PMC6258561

[15] Chen LH, Piyaphanee W, Diaz-Menendez M, Unplanned healthcare during travel: a descriptive analysis from the GeoSentinel Network [Abstract 1398]. In: Proceedings of the American Society of Tropical Medicine and Hygiene Annual Conference; November 18, 2020; Toronto, Canada. Arlington, VA: American Society of Tropical Medicine and Hygiene.

[16] Barnett ED, Mccarthy A, Coyle CM, Global surveillance of infectious diseases in migrants by the GeoSentinel network [Abstract 110]. In: Proceedings of the International Conference on Migration Health; October 2, 2018: Rome, Italy. Dunwoody, GA: International Society of Travel Medicine.

[17] Schwartz E, Meltzer E, Mendelson M, Detection on four continents of dengue fever cases related to an ongoing outbreak in Luanda, Angola, March to May 2013. Euro Surveill 2013;18:20488. 10.2807/ese.18.21.20488-en 23725977

[18] CDC. Ongoing dengue epidemic—Angola, June 2013. MMWR Morb Mortal Wkly Rep 2013;62:504–7. 23784016 PMC4604895

[19] Chen LH. Zika virus infection in a Massachusetts resident after travel to Costa Rica: a case report. Ann Intern Med 2016;164:574–6. 10.7326/L16-0075 26864175

[20] Hamer DH, Barbre KA, Chen LH, ; GeoSentinel Surveillance Network. Travel-associated Zika virus disease acquired in the Americas through February 2016: a GeoSentinel analysis. Ann Intern Med 2017;166:99–108. 10.7326/M16-1842 27893080

[21] Hamer DH, Angelo K, Caumes E, Fatal yellow fever in travelers to Brazil, 2018. MMWR Morb Mortal Wkly Rep 2018;67:340–1. 10.15585/mmwr.mm6711e1 29565840 PMC5868208

[22] World Health Organization. Yellow fever—Brazil. Geneva, Switzerland: World Health Organization; 2019. https://www.who.int/emergencies/disease-outbreak-news/item/18-april-2019-yellow-fever-brazil-en

[23] Lips P, de Jongh RT. Vitamin D deficiency in immigrants. Bone Rep 2018;9:37–41. 10.1016/j.bonr.2018.06.001 30591925 PMC6303232

[24] Dhavan P, Dias HM, Creswell J, Weil D. An overview of tuberculosis and migration. Int J Tuberc Lung Dis 2017;21:610–23. 10.5588/ijtld.16.0917 28482955

[25] Castelli F, Sulis G. Migration and infectious diseases. Clin Microbiol Infect 2017;23:283–9. 10.1016/j.cmi.2017.03.012 28336382

[26] McAuliffe M, Triandafyllidou A, eds. World migration report 2022. Geneva, Switzerland: International Organization for Migration; 2021. https://publications.iom.int/books/world-migration-report-2022

[27] Leder K, Torresi J, Libman MD, ; GeoSentinel Surveillance Network. GeoSentinel surveillance of illness in returned travelers, 2007–2011. Ann Intern Med 2013;158:456–68. 10.7326/0003-4819-158-6-201303190-00005 23552375 PMC4629801

[28] Conner B. Travelers’ diarrhea [Chapter 2]. In: Brunette GW, Nemhauser JB, eds. CDC yellow book 2020: health information for international travel. New York, NY: Oxford University Press; 2019.

[29] Stoney RJ, Han PV, Barnett ED, Travelers’ diarrhea and other gastrointestinal symptoms among Boston-area international travelers. Am J Trop Med Hyg 2017;96:1388–93. 10.4269/ajtmh.16-0447 28719282 PMC5462577

[30] Leder K, Torresi J, Libman MD, ; GeoSentinel Surveillance Network. GeoSentinel surveillance of illness in returned travelers, 2007–2011. Ann Intern Med 2013;158:456–68. 10.7326/0003-4819-158-6-201303190-00005 23552375 PMC4629801

[31] Vila J. New molecular diagnostic tools in traveller’s diarrhea. J Travel Med 2017;24(suppl_1):S23–8. 10.1093/jtm/taw071 28520995

[32] Diemert DJ. Prevention and self-treatment of traveler’s diarrhea. Clin Microbiol Rev 2006;19:583–94. 10.1128/CMR.00052-05 16847088 PMC1539099

[33] Walz EJ, Volkman HR, Adedimeji AA, Barriers to malaria prevention in US-based travellers visiting friends and relatives abroad: a qualitative study of West African immigrant travellers. J Travel Med 2019;26:tay163. 10.1093/jtm/tay163 30602033 PMC6679970

[34] Neave PE, Behrens RH, Jones COH. “You’re losing your Ghanaianess”: understanding malaria decision-making among Africans visiting friends and relatives in the UK. Malar J 2014;13:287. 10.1186/1475-2875-13-287 25064713 PMC4118190

[35] Tan KR, Arguin PM. Malaria [Chapter 4]. In: Brunette GW, Nemhauser JB. CDC yellow book 2020: health information for international travel. New York, NY: Oxford University Press; 2019:267.

[36] Volkman HR, Walz EJ, Wanduragala D, Barriers to malaria prevention among immigrant travelers in the United States who visit friends and relatives in sub-Saharan Africa: a cross-sectional, multi-setting survey of knowledge, attitudes, and practices. PLoS One 2020;15:e0229565. 10.1371/journal.pone.0229565 32163426 PMC7067457

[37] Christaki E. New technologies in predicting, preventing and controlling emerging infectious diseases. Virulence 2015;6:558–65. 10.1080/21505594.2015.1040975 26068569 PMC4720248

[38] Dai Y, Wang J. Identifying the outbreak signal of COVID-19 before the response of the traditional disease monitoring system. PLoS Negl Trop Dis 2020;14:e0008758. 10.1371/journal.pntd.0008758 33001985 PMC7553315

