# *Erratum:* Vol. 72, No. SS-7

**DOI:** 10.15585/mmwr.mm7232a6

**Published:** 2023-08-11

In the Surveillance Summary, “Travel-Related Diagnoses Among U.S. Nonmigrant Travelers or Migrants Presenting to U.S. GeoSentinel Sites — GeoSentinel Network, 2012–2021,” on page 1, in the listing of author affiliations, number 12 should have read, “**^12^The New York Center for Travel and Tropical Medicine, Weill Cornell Medical College, New York, New York**.”

